# P-1076. Describing the Clinical Epidemiology of Metallo-β-lactamase–producing (MBL) Carbapenem-resistant Enterobacterales at a Large Health System

**DOI:** 10.1093/ofid/ofaf695.1271

**Published:** 2026-01-11

**Authors:** Shardul N Rathod, Dariusz A Hareza, Kendall Kling

**Affiliations:** Northwestern Memorial Hospital, Chicago, Illinois; Northwestern University Feinberg School of Medicine, Chicago, Illinois; Northwestern University, Chicago, Illinois

## Abstract

**Background:**

Carbapenem-resistant Enterobacterales (CRE) are increasing in incidence since the COVID-19 pandemic and an urgent threat in the United States; approximately 35% of CRE produce a carbapenemase. Metallo-β-lactamase-producing CRE (MBL-CRE), such as the New Delhi Metallo-β-lactamase (NDM), are of particular clinical importance due to their extensive multidrug-resistance and associated increased morbidity and mortality. Our study describes the clinical epidemiology, infection recurrence, and mortality of MBL-CRE at a large health system.

**Figure 1:**
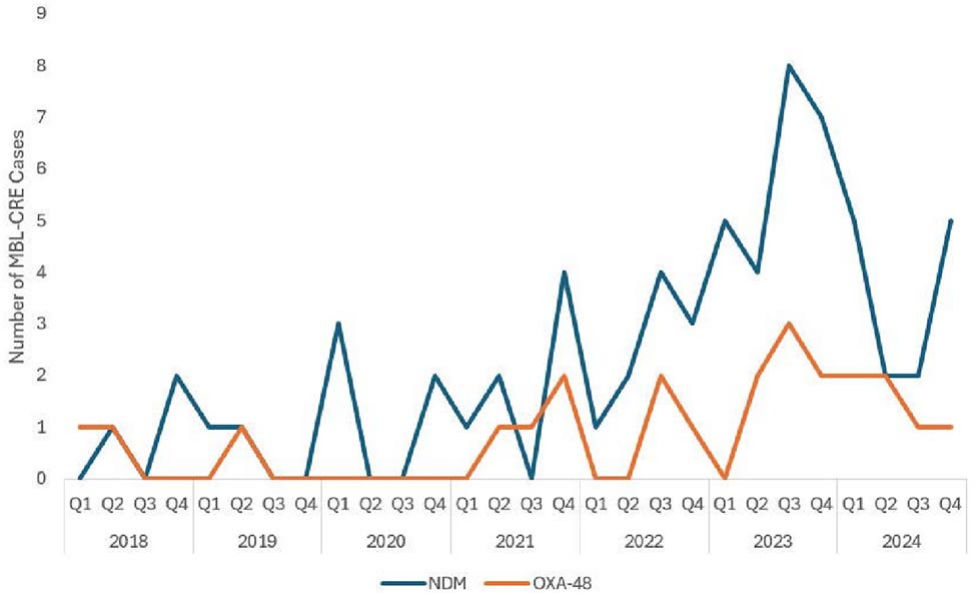
MBL-CRE epidemic curve of patient cases at a large health system from 2018–2024 Abbreviations: MBL-CRE: metallo-β-lactamase-producing carbapenem-resistant Enterobacterales; NDM: New Delhi metallo-β-lactamase; OXA-48: oxacillinase-48

**Table 1:**
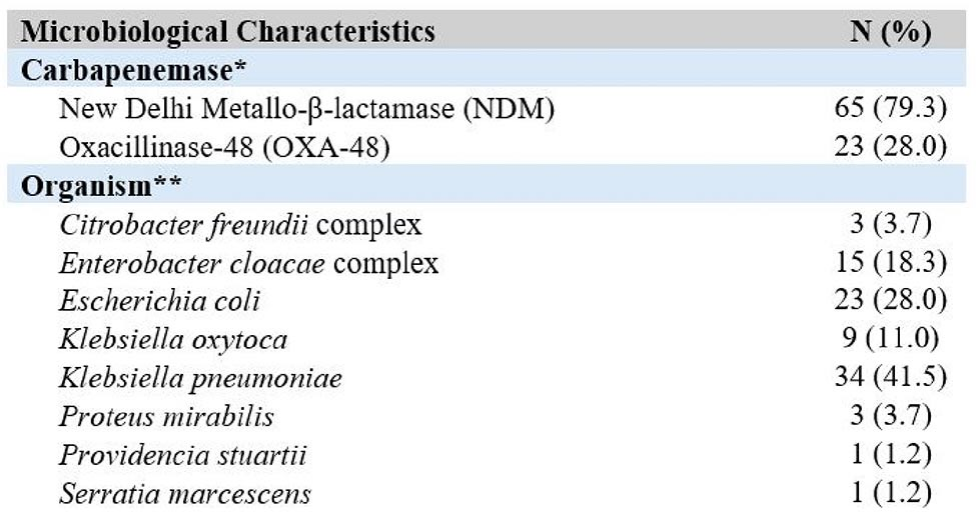
Microbiological characteristics of MBL-CRE isolates in 82 patients at a large health system from 2018–2024 *Some patients (6/82, 7.3%) had isolates that produced more than one carbapenemase enzyme **Some patients (6/82, 7.3%) had more than one organism species produce MBL

**Methods:**

MBL detection was performed by the Xpert® Carba-R assay (Cepheid, Sunnyvale, CA) as part of routine clinical care, and the institutional laboratory archived the relevant isolates. Clinical and epidemiological characteristics were determined through electronic medical record review.

**Table 2:**
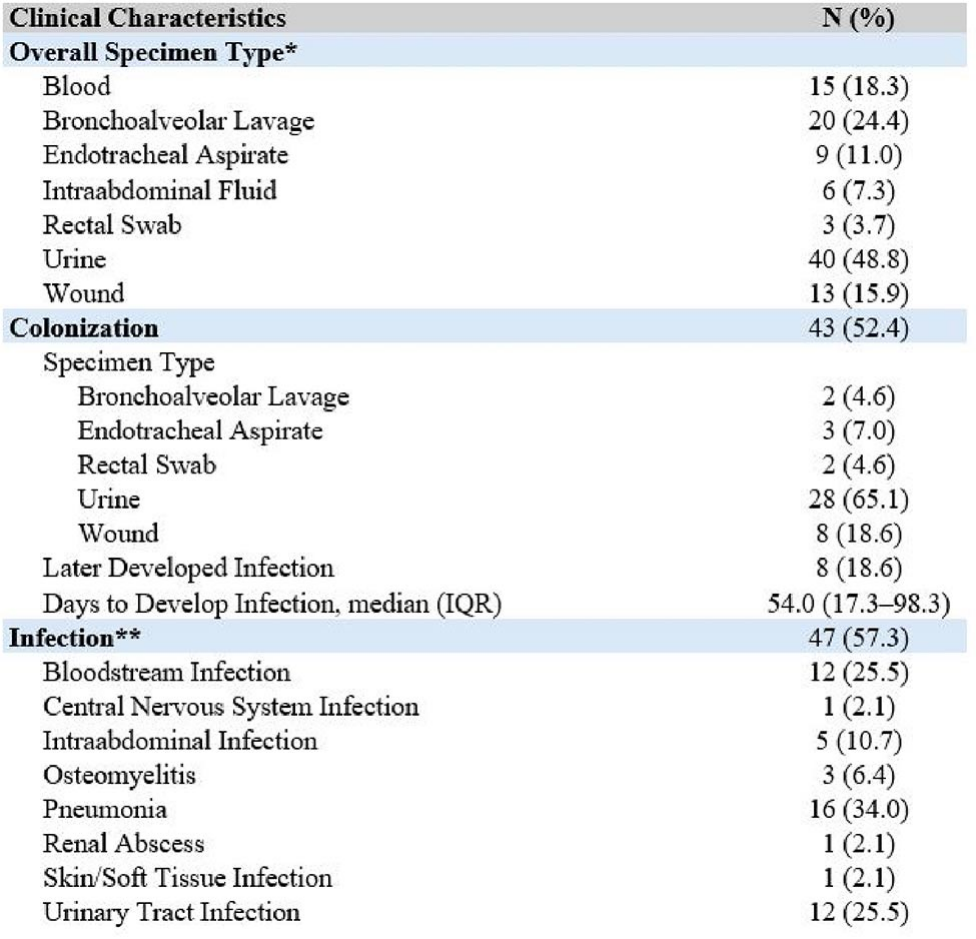
Clinical characteristics of 82 patients colonized or infected with MBL-CRE isolates at a large health system from 2018-2024 *Patients may have had MBL-CRE isolated from more than one specimen type **Includes patients who were initially colonized and subsequently developed infection

**Table 3:**
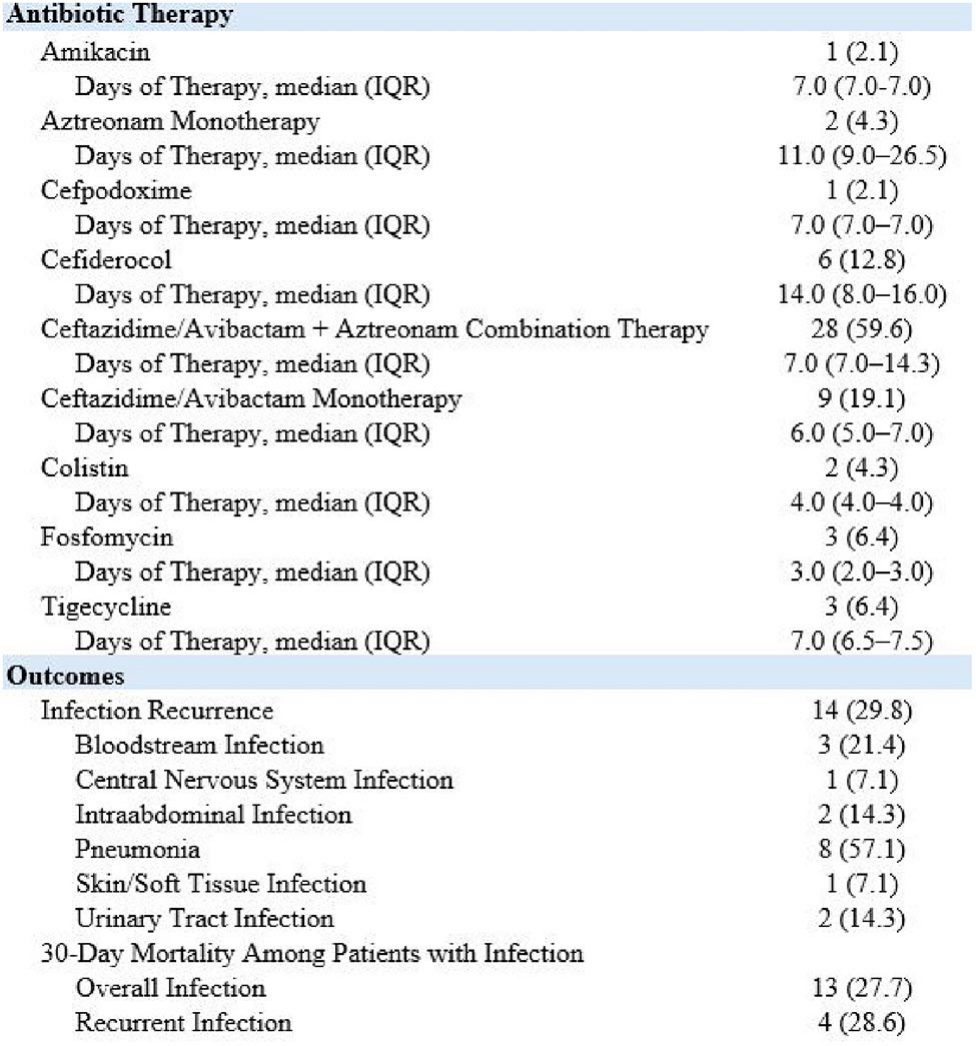
Antibiotic therapy and outcomes of patients with MBL-CRE infections at a large health system from 2018-2024

**Results:**

There were 82 patients with MBL-CRE from 2018–2024. The incidence rose during this time period (NDM: 366.7% increase, p< 0.001; OXA-48: 200.0% increase, p=0.008) (Figure 1). Immunocompromised patients such as those receiving chemotherapy (17.1%) and who underwent solid organ transplantation (18.3%) were commonly represented. NDM (79.3%) was the most common MBL detected (Table 1). Over half the cohort (57.3%) were determined to have infection; 18.6% of patients who were initially colonized subsequently developed infection (Table 2). The three most common types of infections were pneumonia (34.0%), urinary tract infections (25.5%), and bloodstream infections (25.5%). Additionally, 29.8% of patients experienced infection recurrence, defined as a clinically relevant, repeat culture of the same organism and susceptibility pattern after completion of treatment ≥30 days from the first culture (Table 3). There was a 27.7% 30-day mortality among patients with infection.

**Conclusion:**

MBL-CRE commonly affects vulnerable patient populations. The 30-day mortality among patients with infection in our cohort aligns with typically reported data, though our cohort’s infection recurrence is higher than that reported in other CRE studies. Given the rising incidence of MBL-CRE, a better understanding of its clinical epidemiology may better inform empiric treatment, antimicrobial stewardship, and infection prevention practices.

**Disclosures:**

All Authors: No reported disclosures

